# Self-limited membranous nephropathy after intravitreal bevacizumab therapy for age-related macular degeneration

**DOI:** 10.15171/jnp.2017.23

**Published:** 2017-02-05

**Authors:** Gebran Khneizer, Ahmad Al-Taee, Bahar Bastani

**Affiliations:** Department of Internal Medicine, Saint Louis University School of Medicine, Saint Louis, Missouri, USA

**Keywords:** Membranous nephropathy, Proteinuria, Nephrotic syndrome, Intravitreal bevacizumab, Age-related Macular degeneration, Vascular endothelial growth factor, VEGF, Anti-vascular endothelial growth factor, Anti-VEGF

## Abstract

**Background:**

Monoclonal antibodies targeting vascular endothelial growth factor (VEGF), such as bevacizumab, are administered intravitreally for the treatment of wet or exudative age-related macular degeneration (ARMD). Systemic use of bevacizumab has been linked to a wide range of renal adverse effects including proteinuria and hypertension.

**Case Presentation:**

We present the case of a 77-year-old Caucasian male with a past medical history of hypertension, vitamin D deficiency and paroxysmal atrial fibrillation who presented to primary care clinic with a 2-week history of bilateral lower extremity edema, 2 months after completing four monthly intravitreal injections of bevacizumab for ARMD. Examination was remarkable for blood pressure of 187/91 mm Hg and severe bilateral lower extremity edema. Work up revealed unremarkable complete blood count (CBC), comprehensive metabolic panel (CMP), lipid panel, and echocardiography, except for 491 mg/dL albuminuria. Metoprolol and furosemide were added to hydrochlorothiazide and lisinopril. Work up by nephrology consult team five months later was notable for a urinalysis revealing 3 red blood cells/high power field (RBC/HPF), 24-hour urine protein of 8.6 g, and serum creatinine of 1.2 mg/dL. Viral hepatitis panel, total complement activity (CH50), C3, C4, anti-nuclear antibody (ANA), anti-neutrophil cytoplasmic antibody (ANCA), serum and urine protein electrophoresis were all unremarkable. Renal biopsy was consistent with membranous nephropathy. Age-appropriate cancer screening was negative. Random urine protein-to-creatinine ratio declined to 2 g/g and then to 0.56 g/g at 7 and 10 months follow up, respectively. Serum blood urea nitrogen (BUN) and creatinine remained normal throughout the course of illness and patient did not require any immunosuppressive treatment.

**Conclusions:**

The wide range of nephrotoxicity after systemic bevacizumab has been well documented. Our case describes a self-limited biopsy-proven membranous nephropathy after intravitreal bevacizumab injections.

**Implication for health policy/practice/research/medical education:**

Clinicians need to be familiar with the renal side effects of intravitreal anti-VEGF particularly bevacizumab in the setting of growing use of such medications.

## 1. Introduction

Wet or exudative age-related macular degeneration (ARMD) is characterized by choroidal neovascularization with vascular endothelial growth factor-A (VEGF) identified as an essential factor in the pathogenesis of this condition (1). Therefore, targeting the VEGF pathway with various anti-VEGF antibodies is now an established treatment for wet ARMD. For the same reason, anti-VEGF therapy is the mainstay treatment for different primary and metastatic solid cancers as well. There has been a significant dose-dependent increase in risk of hypertension and proteinuria with systemic bevacizumab in cancer patient being treated with such agent (2).

In the past decade, there has been a growing use of intravitreal bevacizumab therapy that has been shown to be associated with renal side effects. Pharmacokinetic studies of intravitreal route of bevacizumab have shown significant systemic levels of the drug achieved after intravitreal administration of the drug (3,4).

Since the majority of data about the renal side effect profile of intravitreal bevacizumab is obtained from uncontrolled sources (5,6), presenting a new case would further our understanding of such side effects.

We present the case of a self-limited biopsy-proven membranous nephropathy after intravitreal bevacizumab therapy used for treatment of wet ARMD.

## 2. Case Presentation

**Figure 1:**
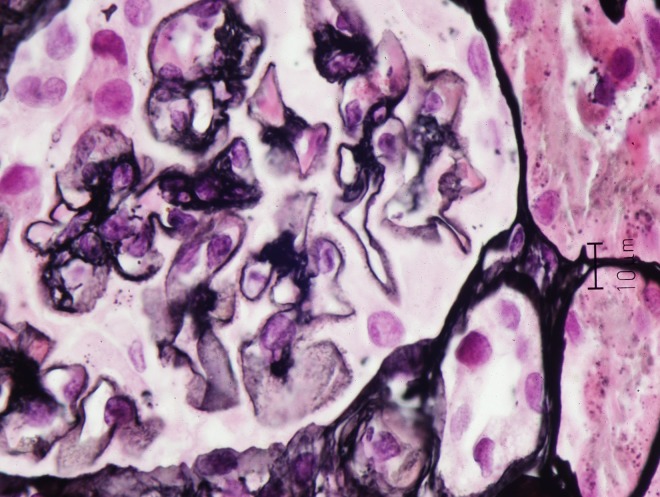
Silver-Jones stain of renal biopsy shows mild focal increase in mesangial matrix, capillary basement membrane is thickened with spikes characteristic of membranous nephropathy (×400).

**Figure 2:**
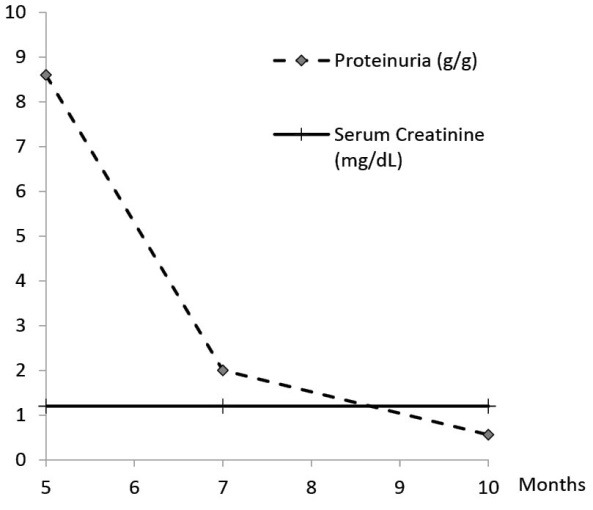
Proteinuria and serum creatinine as a function of months after presentation

A 77-year-old Caucasian male with a past medical history of hypertension, vitamin D deficiency and paroxysmal atrial fibrillation presented to primary care clinic with a 2-week history of bilateral lower extremity edema, two months after completing 4 monthly intravitreal injections of bevacizumab for ARMD. Past surgical history was remarkable for a right hip replacement. Home medications included hydrochlorothiazide, cholecalciferol and lisinopril with no known drug allergies. Social history was remarkable for a 30-pack-year tobacco smoking, and no alcohol or illicit drug use. Vital signs were notable for a blood pressure of 187/91 mm Hg. Physical examination revealed significant bilateral lower extremity edema up to the thighs. Work up which included complete blood count (CBC), comprehensive metabolic panel (CMP), lipid panel, and echocardiography returned within normal limits, except for albuminuria of 491 mg/dL. Metoprolol and furosemide were added to patient’s medication regimen. Five months after presentation, patient was referred to nephrology clinic and laboratory testing was notable for urinalysis revealing 3 red blood cells/high power field (RBC/HPF), 24-hour urine protein of 8.6 g, and serum creatinine of 1.2 mg/dL. A percutaneous renal biopsy revealed mild focal increase in mesangial matrix, minimal interstitial fibrosis, scattered lipid droplets in proximal tubular epithelial cells and on silver-jones staining capillary basement membrane was thickened with spikes. On immunofluorescence, there was 3+ diffuse global granular glomerular basement membrane staining. On electron microscopy, there was global basement membrane thickening with subepithelial electron-dense deposits (immune complexes) with intervening basement membrane (spikes), widespread visceral epithelial foot process effacement, and a mild increase in mesangial matrix. The findings were consistent with membranous nephropathy (Figure 1). Viral hepatitis panel, total complement activity (CH50), C3, C4, anti-nuclear antibody (ANA), anti-neutrophil cytoplasmic antibody (ANCA), serum and urine protein electrophoresis were all within normal limits. Computerized tomography (CT) scan of the chest and colonoscopy were unremarkable, and prostate-specific antigen (PSA) was elevated at 7.5 ng/mL. During follow up, random urine protein-to-creatinine ratio declined to 2 g/g and later to 0.56 g/g at 7 and 10 months, respectively (Figure 2) with subsequent improvement in his lower extremity edema. Serum blood urea nitrogen (BUN) and creatinine remained within normal range throughout the course of illness. Patient did not require any treatment with steroids or immunosuppressant therapy.

## 3. Discussion

VEGF plays an integral role in angiogenesis which is essential for cancer growth and metastasis. In the kidneys, VEGF, which is secreted by podocytes, is essential for optimal functioning of the glomerular filtration barrier (7). In addition, the glomerular capillaries express abundant VEGF receptors (1). Shulman et al documented reduced expression of VEGF in renal biopsies from patients with a known diagnosis of membranous glomerulopathy (8). Altering VEGF pathway has been shown to affect glomerular integrity and function leading to proteinuria and hypertension. These renal side effects have been well described after systemic administration of bevacizumab (2,9).

Pharmacokinetic studies of intravitreal bevacizumab in animals have shown significant systemic levels of the drug. The maximum serum concentration of bevacizumab was reached at day 8 after intravitreal injection (3,4). Taking all this together, it has been suggested that proteinuria can be even used as a surrogate marker for efficacy of VEGF targeting agents (10). Two prior systematic reviews have concluded that cardiovascular and neurological adverse effects are most frequently observed after intravitreal Bevacizumab (11,12). Increase in blood pressure is the most common systemic adverse event, followed by cerebrovascular events and myocardial infarction. Therefore, most literature that describes the side effects of intravitreal bevacizumab focused on hypertension with paucity of reports on renal involvement (12).

The renal side effect of the intravitreal route of bevacizumab remains under-recognized and under-reported (12). The under-recognition of intravitreal bevacizumab renal side effects can be related to a number of factors. First, serum creatinine can remain normal despite significant proteinuria and even nephrotic syndrome similar to our case. Therefore, relying on serum creatinine alone can be falsely reassuring of intact renal function. Second, significant edema or proteinuria can occur several months after administration of intravitreal bevacizumab, which may lead to failure to detect the temporal association. The above points were evident in our case as renal pathology was not pursued early in the evaluation of our patient. The under-reporting of such renal toxicities can also be attributed to the off-label use of intravitreal bevacizumab (13) for management of a variety of ophthalmologic conditions including ARMD. In addition, obtaining medication history does not often involve inquiring about intravitreally-administered agents. Roth et al documented physicians under-reporting such side effects (14). The lack of a specific protocol for intravitreal bevacizumab therapy for ocular conditions - manifested by the wide variation in the number and timing of injections (13) - further impedes the ability to accurately identify the range of such renal side effects.

Micieli et al have suggested a system of surveillance for adverse effects of intravitreal bevacizumab in the form of an internet-based patient registry (11). Such structure involves both physicians and patients entering demographic data, vital signs, and laboratory data at baseline and at specific follow-up time periods. Establishing such registry is especially important in the setting of the growing use of intravitreal bevacizumab in the field of ophthalmology.

In our review of the literature, the renal pathology post intravitreal bevacizumab has been documented in the form of case reports. Anto et al have described a biopsy-proven membranous nephropathy which occurred six months after completing 3 injections of intravitreal bevacizumab for ARMD (15). In that case, immunosuppressive therapy consisting of a 6-month course of oral prednisone and cyclophosphamide was given. Relapse of minimal change nephrotic syndrome after intravitreal bevacizumab has been documented as well (16,17). A pediatric patient with steroid sensitive minimal change disease and multiple prior relapses developed severe myopic choroidal neovascularization requiring intravitreal bevacizumab. The patient developed a new relapse 9 days after the intravitreal therapy and required a long-term course of steroid therapy for multiple ensuing relapses (16). In another case of a 54-year-old male with a history of nephrotic syndrome due to biopsy-proven minimal change disease and four prior relapses 10 years earlier, a new relapse of nephrotic syndrome developed 2 weeks after receiving 2 intravitreal injections of bevazicumab for branch retinal vein occlusion (17).

Our case highlights two principal points. It describes the second biopsy-proven membranous nephropathy occurring after receiving intravitreal bevacizumab injection. More importantly, it resolved spontaneously months after cessation of intravitreal bevacizumab without the need for immunosuppressive therapy.

## 4. Conclusions

The presented case emphasizes the need for clinicians to be diligent in looking for renal disease in patients receiving intravitreal anti-VEGF therapy.

## Acknowledgements

Parts of this paper will be presented in a poster format in the American College of Physicians: Missouri chapter annual meeting on Saturday, Sept 17, 2016.

The authors thank Dr. David Brink, the nephropathologist at Saint Louis University Hospital, for reviewing the pathology slides.

## Authors’ contribution

All authors contributed equally to the preparation of the case report.

## Conflicts of interest

The authors declare no conflict of interest.

## Funding/Support

There was no source of funding for this publication.

## References

[1] Ferrara N (2004). Vascular endothelial growth factor: basic science and clinical progress. Endocr Rev.

[2] Zhu X, Wu S, Dahut WL, Parikh CR (2007). Risks of proteinuria and hypertension with bevacizumab, an antibody against vascular endothelial growth factor: systematic review and meta-analysis. Am J Kidney Dis.

[3] Bakri SJ, Snyder MR, Reid JM, Pulido JS, Singh RJ (2007). Pharmacokinetics of intravitreal bevacizumab (Avastin). Ophthalmology.

[4] Sinapis CI, Routsias JG, Sinapis AI, Sinapis DI, Agrogiannis GD, Pantopoulou A (2011). Pharmacokinetics of intravitreal bevacizumab (Avastin(R)) in rabbits. Clin Ophthalmol.

[5] Wu L, Martinez-Castellanos MA, Quiroz-Mercado H, Arevalo JF, Berrocal MH, Farah ME (2008). Twelve-month safety of intravitreal injections of bevacizumab (Avastin): results of the Pan-American Collaborative Retina Study Group (PACORES). Graefes Arch Clin Exp Ophthalmol.

[6] Fung AE, Rosenfeld PJ, Reichel E (2006). The International Intravitreal Bevacizumab Safety Survey: using the internet to assess drug safety worldwide. Br J Ophthalmol.

[7] Eremina V, Sood M, Haigh J, Nagy A, Lajoie G, Ferrara N (2003). Glomerular-specific alterations of VEGF-A expression lead to distinct congenital and acquired renal diseases. J Clin Invest.

[8] Shulman K, Rosen S, Tognazzi K, Manseau EJ, Brown LF (1996). Expression of vascular permeability factor (VPF/VEGF) is altered in many glomerular diseases. J Am Soc Nephrol.

[9] Wu S, Kim C, Baer L, Zhu X (2010). Bevacizumab increases risk for severe proteinuria in cancer patients. J Am Soc Nephrol.

[10] Saloustros E, Androulakis N, Vamvakas L, Mavroudis D, Georgoulias V (2010). Favorable clinical course of patients experiencing bevacizumab-induced proteinuria. Case Rep Oncol.

[11] Micieli JA, Micieli A, Smith AF (2010). Identifying systemic safety signals following intravitreal bevacizumab: systematic review of the literature and the Canadian Adverse Drug Reaction Database. Can J Ophthalmol.

[12] van der Reis MI, La Heij EC, De Jong-Hesse Y, Ringens PJ, Hendrikse F, Schouten JS (2011). A systematic review of the adverse events of intravitreal anti-vascular endothelial growth factor injections. Retina.

[13] Gunther JB, Altaweel MM (2009). Bevacizumab (Avastin) for the treatment of ocular disease. Surv Ophthalmol.

[14] Roth DB, King A, Weiss M, Klein D (2009). Systemic adverse events after bevacizumab. Ophthalmology.

[15] Anto HR, Hyman GF, Li JP, Spitalewitz S, Thomas D (2012). Membranous nephropathy following intravitreal injection of bevacizumab. Can J Ophthalmol.

[16] Sato T, Kawasaki Y, Waragai T, Imaizumi T, Ono A, Sakai N (2013). Relapse of minimal change nephrotic syndrome after intravitreal bevacizumab. Pediatr Int.

[17] Perez-Valdivia MA, Lopez-Mendoza M, Toro-Prieto FJ, Cabello-Chaves V, Toro-Ramos M, Martin-Herrera MC (2014). Relapse of minimal change disease nephrotic syndrome after administering intravitreal bevacizumab. Nefrologia.

